# Prevalence of Chronic Polypharmacy in Community-Dwelling Elderly People in Poland: Analysis of National Real-World Database Helps to Identify High Risk Group

**DOI:** 10.3389/fphar.2021.739740

**Published:** 2021-11-18

**Authors:** Przemysław Kardas, Aneta Lichwierowicz, Filip Urbański, Ewa Chudzyńska, Marcin Czech, Grzegorz Kardas

**Affiliations:** ^1^ Department of Family Medicine, Medical University of Lodz, Łódź, Poland; ^2^ National Health Fund, Warsaw, Poland; ^3^ Department of Pharmacoeconomics, Institute of Mother and Child, Warsaw, Poland; ^4^ Department of Internal Diseases, Asthma and Allergy, Medical University of Lodz, Łódź, Poland

**Keywords:** polypharmacy, potentially inappropriate prescribing, drug safety, elderly, pharmacoepidemiology, real-word data, healthcare services utilization, Poland

## Abstract

**Introduction:** Multimorbidity often comes with age, making elderly people particularly prone to polypharmacy. Polypharmacy, in turn, is a risk factor for adverse drug reactions, drug-drug interactions, non-adherence to medication, negative health outcomes, and increased healthcare services utilization. The longer the exposure to polypharmacy is, the higher the risk of these consequences is. Therefore, a detailed assessment of the prevalence and drivers of chronic polypharmacy in the elderly is particularly important.

**Aim of study:** To find out the prevalence of chronic polypharmacy in the elderly population of Poland, and to characterize the subgroup with the highest risk of this problem, using real-world data.

**Methodology:** A retrospective analysis of data on dispensation and healthcare services utilization held by the national payer organization for the year 2018. Chronic polypharmacy was defined as possession, as a result of dispensation, of five or more prescribed drugs within 80% of each of the consecutive 6 months.

**Results:** Chronic polypharmacy was found in 554.1 thousand patients, i.e. in 19.1% of the national 65+ cohort. On average, those patients were 76 years old, and 49.3% of them were female. The vast majority (68.6%) continued their polypharmacy for the period of the whole year. There was a marked variation in geographical distribution of chronic polypharmacy with the highest value of 1.7 thousand per 100,000 inhabitants in the Łódź Voivodeship. Patients exposed to chronic polypharmacy filled prescriptions from 4.5±2.36 healthcare professionals. The average number of drugs they used was 8.3±3.84 DDD per patient per day. The most often prescribed drugs were Metformin, Atorvastatin and Pantoprazole. The average annual hospitalisation rate in those patients was 1.03±2.4.

**Conclusion:** This study was the first of this kind involving a nationwide assessment of chronic polypharmacy in Polish elderly people. We found that this problem affected one fifth of Polish older adults and it remains stable due to its direct relation to chronic conditions. Thus, our results confirm that this phenomenon is highly important for the national health policy and requires relevant interventions. The planned introduction of pharmaceutical care in Poland is expected to help in solving the problem.

## Introduction

Twenty-first century medicine is witnessing an unprecedented paradox. On the one hand, achievements of modern medicine and pharmacy, along with a boost in global economy, led to a new scenario offering billions of human beings access to medications they need. On the other hand, multiple medicines used by a lot of individuals not only add to the spiral rise of healthcare costs but also entail additional risks. Thus, a positive concept of ‘access to drugs in case of need’ is slowly evolving into an unfavourable phenomenon of “polypharmacy”, which becomes a chronic problem, particularly among the elderly and constitutes one of the major concerns of the public health worldwide.

Polypharmacy lacks a standard consensual definition. Nevertheless, its paradoxical nature is reflected in terminology. In general, polypharmacy describes a scenario in which multiple medicines are used by the same patient. In practical terms, polypharmacy is most often defined as a concurrent use of five or more drugs, whereas the use of ten or more drugs is usually regarded as ‘excessive polypharmacy’ (World Health Organization, 2019). However, these numbers are used for pragmatic reasons only as there is no firm evidence based on which a sound threshold could be created, dichotomizing the number of drugs used concurrently to be either acceptable or too high (Masnoon et al., 2017).

Risks associated with polypharmacy are multiple and include potentially inappropriate prescribing, adverse drug reactions, drug-drug interactions and decreased adherence to medication, which all lead to further negative health consequences, such as reduced quality of life, negative health outcomes and increased healthcare services utilisation (Khezrian et al., 2020; Lee et al., 2020). The longer the exposure to polypharmacy is, the more probable and more pronounced all of these negative consequences are.

The significance of the challenge created by polypharmacy has been increasing, especially due to its escalating prevalence. Particularly in Europe, recent years have seen a growing trend for this phenomenon, e.g., in Sweden the prevalence of polypharmacy increased between 2006 and 2014, from 16.9 to 19.0% (Zhang et al., 2020).

While polypharmacy might be temporal among younger patients (e.g. due to infection), it is usually chronic in the elderly. The possibility of long-term multidrug therapies increases with age and related multimorbidity. These interlinked factors are undoubtedly the major drivers of the recent rise in polypharmacy prevalence in Europe. Data collected in the UK showed that 20.8% of patients with two clinical conditions were prescribed four to nine medicines, whereas in those with six or more comorbidities, the relevant percentage was 47.7%. At the same time, statistics show that more than 50% of people aged over 65 years are diagnosed with two or more diseases, and the older they get, the more diseases they suffer from (Barnett et al., 2012).

Therefore it should come as no surprise that the problem of polypharmacy in elderly people is already widespread in Europe. A nationwide cohort study conducted in Sweden among individuals aged ≥65 years found prevalence of polypharmacy in 44.0% of the group, and the prevalence of excessive polypharmacy in 11.7% (Morin et al., 2018). In this country, the existence of these scenarios strictly correlates with age and peaks up to 79.6 and 36.4% in individuals aged 90 years and over, respectively (Zhang et al., 2020). Scottish data provide evidence that around 35% of those aged 85 years and over receive more than ten medicines (Stewart et al., 2017). A recent analysis of a large European cohort found polypharmacy to occur in 32.1% of citizens aged 65 years or above, ranging from 26.3 to 39.9% across 18 of the studied countries (Midão et al., 2018).

The observed rise in polypharmacy prevalence is particularly pronounced in the elderly (Hovstadius et al., 2010). Over the period of 15 years, the prevalence of polypharmacy in this group of patients grew fourfold in Ireland, from 17.8 to 60.4% (Moriarty et al., 2015). Currently those aged over 65 years account for 19.2% of the European Union population, and this proportion is expected to reach 29.1% by 2080, whereas in the case of those aged over 80 years, relevant percentage rates will change from the present 5.4–12.7% (Eurostat, 2020). If these predictions prove to be true, the burden of polypharmacy is expected to rise dramatically in Europe in the upcoming decades.

In Poland, neither prevalence nor characteristics of polypharmacy have been studied extensively so far. Nevertheless, there are good arguments to believe that its significance might particularly result from high use of prescription medications in the country. In the European health interview survey (EHIS), the collected data indicated a slightly higher use of such medications in Poland, as compared to the EU-28. That tendency, however, was particularly pronounced in the elderly–use of prescription medications was reported by 83.6% of those aged 65–74 years, and 92.8% of those aged 75 years and over in Poland, whereas the European average in these groups was 78.1%, and 87.1%, respectively (Eurostat, 2021). Moreover, a very high prevalence of polypharmacy (78.6%) was observed among Polish patients provided with palliative care (Grądalski, 2019).

Moreover, the Polish population is ageing fast (Leszko et al., 2015). Consequently, polypharmacy has become a serious medical, social and economic threat, exerting a major impact on the sustainability of the national healthcare system. From this perspective, an objective assessment of polypharmacy rates in elderly citizens is of utmost importance for the national health policy. What is of particular interest, however, is the identification of the most vulnerable group of patients being at high risk of chronic polypharmacy.

The aim of our study was to estimate the prevalence of chronic polypharmacy in the elderly (65+) population in Poland, using databases of the National Health Fund (NHF, Polish: *Narodowy Fundusz Zdrowia*). NHF is the sole public payer organization in the Polish healthcare system with a nationwide coverage. The NHF databases collect information on dispensation of reimbursed drugs, as well as on utilization of healthcare services. Based on an analysis of the data, we wanted to characterise the subgroup of elderly patients with the highest risk of chronic polypharmacy.

## Methodology

### Data and Study Design

This was a retrospective analysis of data on drug dispensation and healthcare services utilization recorded in NHF databases for 2018.

The NHF databases register full information on dispensation of all drugs which are subject to reimbursement, regardless of whether a particular prescription was issued by a public or a private healthcare provider. Thus, according to availability of data, we studied prevalence of polypharmacy caused by dispensation of reimbursed drugs only. Data available for the analysis of healthcare services utilization included the type and number of both hospitalisations as well as ambulatory services, along with their principal diagnoses.

In order to avoid a bias of short-term therapies of no importance for chronic treatment, the analysis excluded medications from the following Anatomical Therapeutic Chemical (ATC) groups: A01 - Stomatological preparations, A06 - Drugs for constipation, D–Dermatologicals, J01 - Antibacterials for systemic use, J02 - Antimycotics for systemic use, J05 - Antivirals for systemic use, J06 - Immune sera and immunoglobulins, J07–Vaccines, P03 - Ectoparasiticides, including scabicides, insecticides and repellents and V–Various.

### Definition of Polypharmacy

For the purpose of this study, polypharmacy was operationalised as taking five or more prescribed medications at the same time, according to the most common approach, as suggested by the WHO report (World Health Organization, 2019). A 6-month period was adopted a basic framework for the analysis. Accordingly, relevant numbers of drugs were calculated according to the number of reimbursed drugs dispensed within 6 months from the first dispensation in the calendar year.

Chronic polypharmacy was operationalised as “possession, as a result of dispensation, of five or more prescribed drugs within each of consecutive 6 months” where “possession” meant at least 80% of a period covered by dispensed drug supply. For calculation of daily doses, the WHO standard daily defined doses (DDDs) were used.

### Statistical Analyses

In descriptive statistics, both original numbers, means, medians and standard deviations, as well as the percentage rates calculated out of the total number of identified polypharmacy cases were presented, unless otherwise stated. For calculation purposes, the national population of Poland in 2018 was assumed to be 38,411,148, and a number of citizens aged over 65 years to be 6,732,360, according to public statistics (Statistics Poland, 2019).

### Ethics

Analyses of aggregated anonymised dispensation data and health services utilisation do not involve ethical issues. Therefore, according to the policy of the Ethical Commission of the Medical University of Lodz, those analyses were not subject to the ethical approval procedure.

## Results

### Prevalence of Polypharmacy in the Elderly

Among all Polish citizens who satisfied our operational definition of polypharmacy (i.e. were dispensed ≥5 reimbursed drugs within 6 months from their first dispensation in 2018, 4.507M in total), those aged over 65 years accounted for 2.899M, i.e. 64.3%. It means that 43.1% of Polish elderly people were subject to polypharmacy in 2018.

**FIGURE 1:**
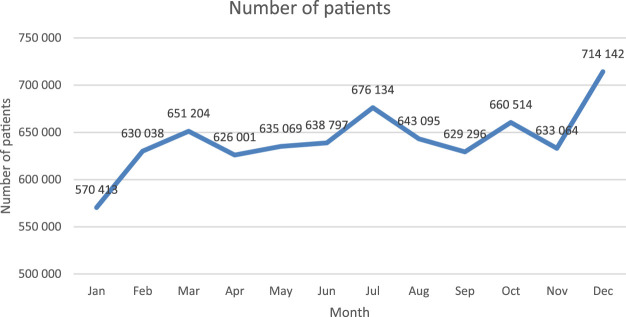
Number of Polish elderly patients who during at least 80% of days within a specific month of year 2018 were on five or more drugs.

The number of elderly patients on polypharmacy who had five or more prescribed drugs for at least 80% of days in a month varied over the time, being the highest in December 2018, and the lowest in January 2018 (Figure 1).

Within the group, 554.1 thousand individuals used ≥5 reimbursed drugs for at least 80% of days in half-year horizon, satisfying our definition of chronic polypharmacy. Those individuals accounted for 1.4% of the total national population, and 19.1% of the national cohort of those aged over 65 years.

### Characteristics of Elderly on Chronic Polypharmacy

**FIGURE 2:**
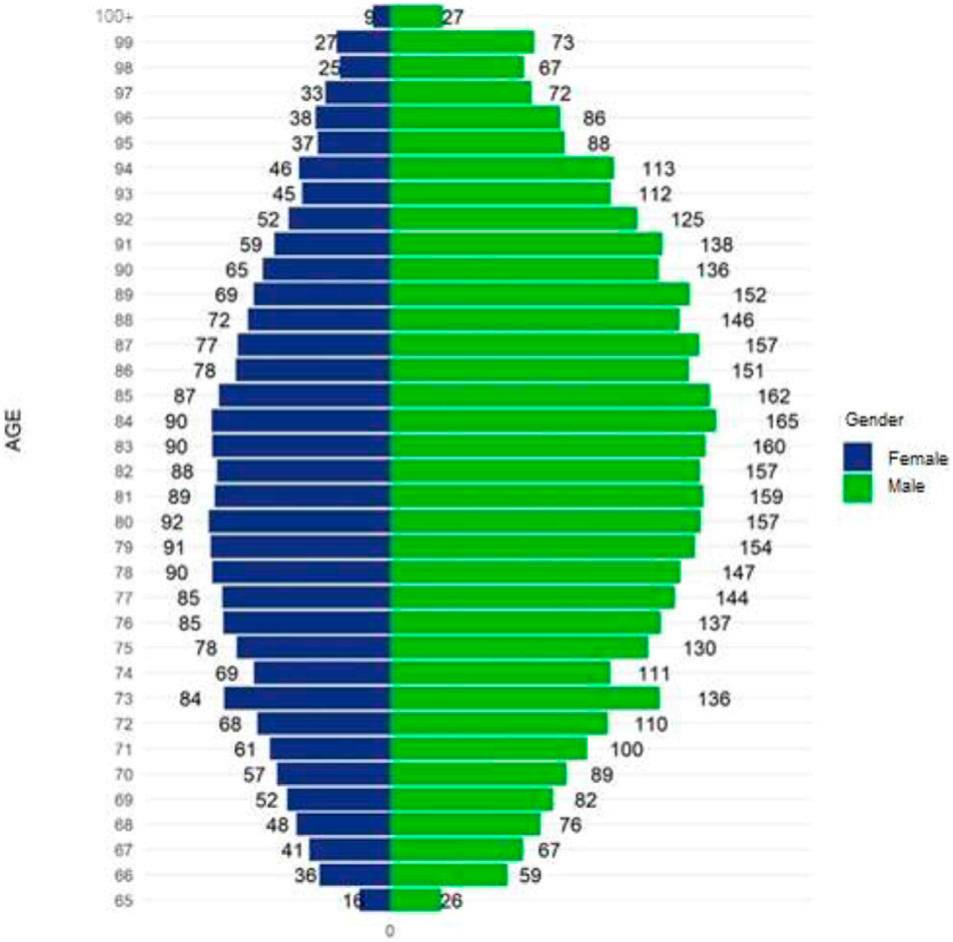
Number of elderly patients on chronic polypharmacy in Poland in 2018 per 1,000 of inhabitants by age and gender.

Detailed characteristics of a group of elderly patients on chronic polypharmacy are presented in Table 1. On average, those patients were 76 years old, and 49.3% of them were female. The proportion of men on chronic polypharmacy was highest for those aged 84 years (16.5%), whereas in women it was generally lower, with the peak for the age of 83–84 years (9.0%) (for details, see Figure 2).

**TABLE 1:** Detailed characteristics of a group of Polish elderly patients who were identified to be a subject of chronic polypharmacy in 2018.

Parameter		N	%
Elderly people on chronic polypharmacy		554,085	100.0
Individuals alive as of December 31, 2018		532,218	96.1
Gender	Male	272,922	49.3
	Female	259,263	46.8
	Missing data	33	0.0
Age (years)	65–69	116,091	21.0
	70–74	132,378	23.9
	75–79	113,440	20.5
	80–84	99,269	17.9
	85–89	54,077	49.3
	90–94	15,006	46.8
	95+	1 957	0.0

**FIGURE 3:**
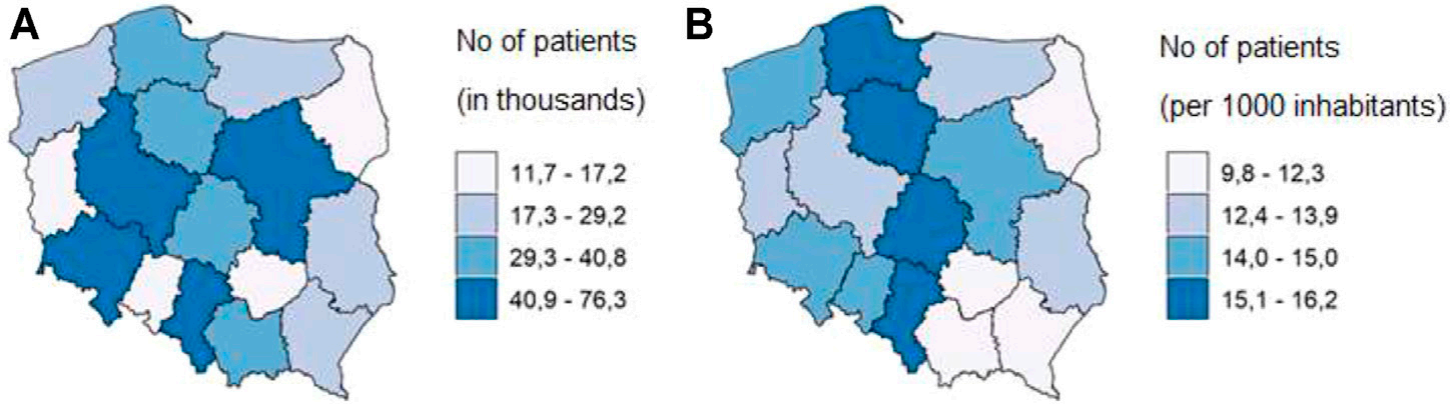
Number of elderly patients on chronic polypharmacy in Poland in 2018 by Voivodeship **(A)**, and per 1,000 inhabitants of Voivodeship **(B)**.

There was a marked variation in geographical distribution of chronic polypharmacy among Polish elderly people. First of all, patients affected by this phenomenon lived either in Poland’s capital city, Warsaw (30,0 k, 5.4%), or in the third most populated Polish city, i.e. Łódź (14,8 k, 2.7%). Per 1,000 inhabitants of the county (Polish: powiat), the highest percentage of patients was observed in Sosnowiec county - 23 per 1,000 inhabitants, and in Łódź county - 22 per 1,000 inhabitants, whereas the lowest one was found in the Leżajsk county - 6.5 per 1,000 inhabitants. In terms of the number of inhabitants of the Voivodeship, the greatest number of people with chronic polypharmacy was observed in the Łódź Voivodeship - 1.7 thousand per 100 thousand inhabitants, and the lowest one in the Podkarpackie Voivodeship - 1.0 thousand per 100 thousand inhabitants (Figure 3).

Out of the total number of elderly patients on chronic polypharmacy, the vast majority (0.380M, i.e. 68.6%) continued their polypharmacy for the period of the whole year.

### Prescriptions Contributing to Chronic Polypharmacy

**FIGURE 4:**
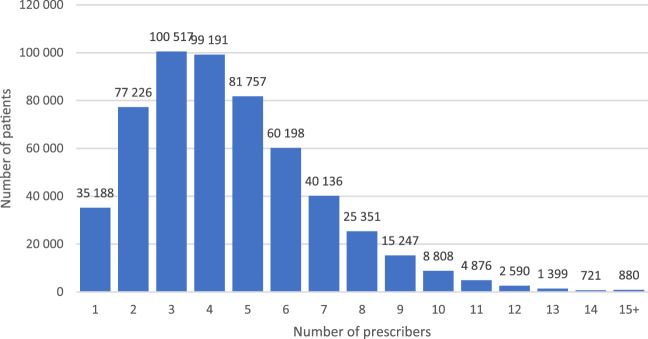
Number of elderly patients on chronic polypharmacy in Poland in 2018 by number of prescribers who issued their prescriptions.

**FIGURE 5:**
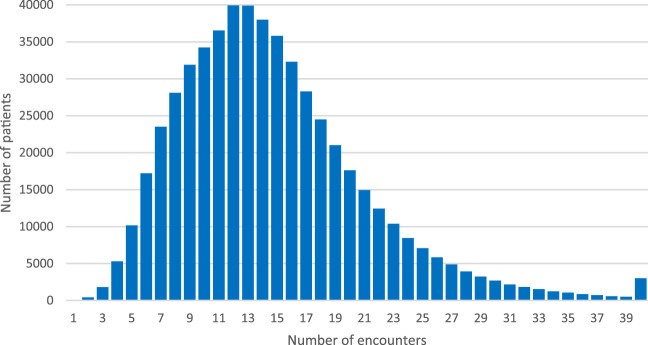
Number of elderly patients on chronic polypharmacy in Poland in 2018 by number of encounters during which their prescriptions were issued.

Patients exposed to chronic polypharmacy filled prescriptions on average from median 4 (mean: 4.5±2.36) healthcare professionals (Figure 4) issued at 14 (mean 14.7 ±6.61) medical encounters in the year 2018 (Figure 5). Almost 30% (28.9% - 160.2 thousand) of patients on chronic polypharmacy in 2018 filled prescriptions from six or more prescribers (another prescriber every 2 months, on average). Approximately, 1% of this group (5.6 k) filled their prescriptions from another prescriber each month, on average.

**FIGURE 6:**
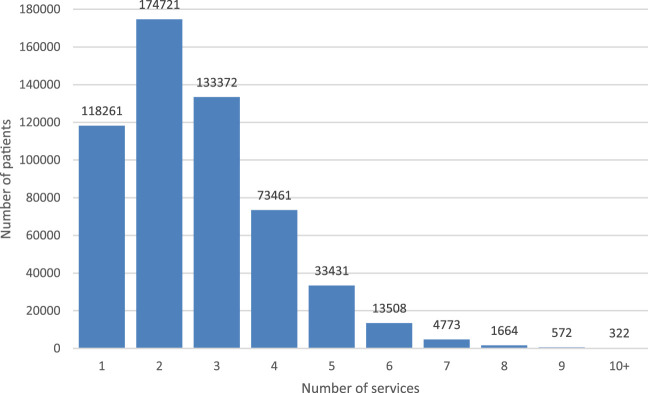
Number of elderly patients on chronic polypharmacy in Poland in 2018 by number of healthcare services in which their prescriptions were issued.

During the year, the average number of medical institutions in which a prescription was issued for them was 2.6±1.39 (Figure 6). In the group of the analysed patients, 23% (127.7 thousand) filled prescriptions from over three service providers, and 1% (7.3 thousand) filled prescriptions from seven or more service providers in 2018. The maximum number of healthcare providers issuing prescriptions per patient per year was 32.

Patients on chronic polypharmacy were dispensed 13.6 million prescriptions (9.1% of all prescriptions for ready-made drugs) for 49.0 million packages of drugs (12.1% of all packages). The average annual number of dispensed prescriptions per patient was 24.6 (+/−10.3), the average number of dispensed drug packs per patient − 88.4 (+/− 31.6). The average number of DDD was 2 784±1,401 per patient per year, i.e. 8.3±3.84 DDD per patient per day. Out of the total number of patients on chronic polypharmacy, 25% (130,968) took an average of ten or more DDD per day, and 1% (5 164) took 24 or more doses per day.

### Drugs Contributing to Chronic Polypharmacy in Elderly People

Among 20 most common drugs contributing to chronic polypharmacy, the most prevalent were cardiovascular agents (seven purely cardiovascular drugs, as well as doxazosin used in both hypertension and benign prostate hyperplasia (BPH)), oral antidiabetic agents (three 3 drugs), drugs used in BPH (two agents indicated specifically for BPH + doxazosin, see comment above), and lipid lowering drugs (two agents). The most frequently prescribed drugs among those patients were as follows: Metformin (50.3% patients, 13.1% prescriptions), Atorvastatin (46.7% patients, 11.4% prescriptions), Pantoprazole (38.0% patients, 7.1% prescriptions), Ramipril (35,3% patients, 9.0% prescriptions) and Amlodipine (33.2% patients, 8.0% prescriptions) (for details, see Table 2).

**TABLE 2:** Top-20 drugs prescribed to elderly patients with chronic polypharmacy.

No	Medication	% Of patients prescribed particular drug (N = 554,085)	% Of prescriptions (N = 13,607,512)
1	Metformin	50.3	13.1
2	Atorvastatin	46.7	11.4
3	Pantoprazole	38.0	7.1
4	Ramipril	35.3	9.0
5	Amlodipine	33.2	8.0
6	Furosemide	27.2	6.4
7	Allopurinol	25.8	5.5
8	Rosuvastatin	25.3	5.9
9	Tamsulosin	24.7	5.1
10	Nebivolol	23.9	6.0
11	Tramadol + Paracetamol	23.3	3.1
12	Indapamide	23.3	5.6
13	Potassium Chloride	21.9	4.0
14	Finasteride	21.1	4.2
15	Spironolactone	18.5	3.3
16	Omeprazole	18.1	3.4
17	Levothyroxine	16.7	2.9
18	Doxazosin	16.5	3.7
19	Gliclazide	15.9	3.9
20	Glimepiride	14.4	3.8

### Healthcare Services Utilisation by Elderly People on Chronic Polypharmacy

Among patients on chronic polypharmacy, 98.7% used primary care services and 86.3% used ambulatory specialist consultations. The number of primary healthcare consultations was 7.1 million (i.e. 12.8 per patient, on average), and in outpatient specialist care - 4.2 million (7.6 per patient). On average, in 2018 patients received care from 2.6±1.39 different types of healthcare services (Table 3).

**TABLE 3:** Healthcare services utilization by elderly patients on chronic polypharmacy.

Health service	No. of patients utilizing particular healthcare service	% Of patients on chronic polypharmacy utilizing particular healthcare service (N = 554,085)
Primary healthcare	544,934	98.7
Outpatient specialist services	476,681	86.3
Hospital treatment	284,798	51.6
Medical rehabilitation	122,950	22.3
Emergency medical services	118,065	21.4
Psychiatric care and addiction treatment	39,279	7.1
Services contracted separately	14,948	2.7
Palliative and hospice care	8 651	1.6
Nursing and care services	7,749	1.4
Pilot programs	3,008	0.5

After the technical code ‘Appointment for issue of a repeat prescription’ (ICD-10 code: Z76.0), the most frequent diagnosis made in these patients in outpatient settings were primary hypertension (51.0%) and type 2 diabetes (35.5%) (for details, see Table 4).

**TABLE 4:** Top-20 principal diagnoses in selected patients on chronic polypharmacy in 2018 by healthcare services other than hospitalisation.

No	Diagnosis	No. of patients	% Of elderly patients on chronic polypharmacy
1	Repeat prescriptions	316,721	57.2
2	Primary hypertension	282,855	51.0
3	Non-insulin dependent diabetes mellitus	196,833	35.5
4	Prostatic hyperplasia	120,116	21.7
5	Persons encountering health services in other specified circumstances	119,322	21.5
6	Chronic ischemic heart disease	112,948	20.4
7	Persons consulting on behalf of another person	78,269	14.1
8	Heart failure	76,304	13.8
9	Atrial fibrillation and flutter	69,165	12.5
10	Encounter for medical observation for suspected diseases and conditions ruled out	68,894	12.4
11	Degenerative changes of the spine	67,930	12.3
12	Chronic obstructive pulmonary disease	64,994	11.7
13	Acute infection of the upper respiratory tract, multiple or undefined	63,840	11.5
14	Polyosteoarthritis	57,674	10.4
15	Hypertensive disease involving the heart	56,209	10.1
16	Bronchial asthma	53,326	9.6
17	People contacting the health service for consultation and advice other than classified elsewhere	50,564	9.1
18	Disorders of the spinal nerve roots and nerve plexuses	49,639	9.0
19	General medical examination of people without ailments and without disease diagnosis	47,747	8.6
20	Acute bronchitis	46,699	8.4

Out of the entire group, 51.6% patients were hospitalized, mainly due to heart failure (3.9% of patients) (Table 5). The total number of hospitalisations in this group of patients in 2018 was 0.569M, making the average annual hospitalization rate 1.03 ± 2,4 (mean±SD), whereas in the general population of elderly citizens, the relevant number was 0,55± 1,42.

**TABLE 5:** Principal diagnoses of hospitalisations in elderly people on chronic polypharmacy in 2018.

No	Principal diagnosis	No	% Of hospitalisations of elderly patients on chronic polypharmacy (N = 0.569M)	% Of elderly patients on chronic polypharmacy (N = 0.554M)
1	Heart failure, unspecified	21,524	3.8	3.9
2	Complicated cataract	16,775	2.9	3.0
3	Congestive heart failure	16,018	2.8	2.9
4	Atrial fibrillation and flutter	10,752	1.9	1.9
5	Other forms of age-related cataracts	9,508	1.7	1.7
6	Heart and blood vessel disease in the course of atherosclerosis	8 831	1.6	1.6
7	Left ventricular heart failure	6,294	1.1	1.1
8	Spontaneous (primary) hypertension	6,146	1.1	1.1
9	Cancer chemotherapy cycles	6,103	1.1	1.1
10	Acute subendocardial infarction	6,034	1.1	1.1
11	Atherosclerosis of the extremities	4,900	0.9	0.9
12	Unstable angina	4,503	0.8	0.8
13	Age-related nuclear cataract	3,948	0.7	0.7
14	Acute kidney failure, unspecified	3,734	0.7	0.7
15	Chronic ischemic heart disease, unspecified	3,703	0.7	0.7
16	Heart disease in the course of atherosclerosis	3,327	0.6	0.6
17	Acute myocardial infarction, unspecified	3,302	0.6	0.6
18	Non-insulin dependent diabetes mellitus (with renal complications)	3,221	0.6	0.6
19	Unspecified chronic obstructive pulmonary disease in exacerbation	3,092	0.5	0.6
20	Chest pain, unspecified	3,060	0.5	0.6

## Discussion

To the best of the authors’ knowledge, this is the first study to describe chronic polypharmacy in Polish elderly people. It provides detailed characteristics of the subgroup of patients who are most likely to be affected by this phenomenon. Our findings show that chronic polypharmacy is a frequent problem among Polish older adults. On average, over 40% of Polish elderly citizens were on polypharmacy in 2018, and every fifth was on chronic polypharmacy. Considering the fact that the population of Poland is aging fast, and the number of elderly citizens is continuously rising, these findings are crucial for the national health policy, and definitely, deserve a lot of attention.

Despite diverse definitions of polypharmacy and methodologies employed in individual studies, recent European data are also alarming when it comes to the prevalence of polypharmacy in the elderly. In the last years, the prevalence of this phenomenon among older people has risen even further in Europe (Carmona-Torres et al., 2018). Polypharmacy was observed in 21.9% of community-dwelling Spanish elderly people (Carmona-Torres et al., 2018), 39.4% of Italian elderly citizens (Slabaugh et al., 2010), 41.2% of Swiss older adults (Blozik et al., 2013), and 51% of Danish individuals aged over 75 years (Kornholt and Christensen, 2020). Based on the data collected in the Sixth Wave of SHARE survey, completed in November 2015, polypharmacy was identified in 32.1% of elderly Europeans, on average, whereas in Poland, this ratio was higher and amounted to approximately 33.8% (Midão et al., 2018).

Due to its natural background related to multimorbidity which is usually composed of chronic diseases, polypharmacy in older age is in most cases a chronic condition (Muth et al., 2019). This, however, does not mean that its long-term nature is well-studied. One of the few exceptions is a longitudinal nationwide cohort study including all older Swedish adults (aged 65 years and over) with five or more prescription drugs in October 2010. The proportion of individuals who remained exposed to polypharmacy after 6 months, 12 months and until the end of this over 3-years-long study was 82, 74 and 55%, respectively (Wastesson et al., 2019). Our own observations of two thirds of chronic polypharmacy patients who maintain this status for the period of the whole year suggests that once an elderly person is prescribed a high number of drugs, the chances for reducing this number are very low. Another study covered an even longer period of 10 years (between 2000 and 2010) and analysed data of nearly two million patients aged 65–94 years living in Lombardy (Northern Italy). The overall prevalence of chronic polypharmacy, defined as administration of five or more drugs during 1 month for at least six (consecutive or not) months in a year, rose from 1.33% in 2000 to 3.34% in 2005 and 7.10% in 2010 (Franchi et al., 2013). In a Dutch study which included 45,731 patients aged 55 years or over with at least one prescribed medication, 27% were found to experience polypharmacy. The number of medications used in the polypharmacy group was on average 11.2 of which 6.9 was used chronically (Sinnige et al., 2016).

Our findings indicate that with similar absolute numbers, chronic polypharmacy was much more prevalent in Polish elderly men than in women. This is an unexpected result as in the studies performed in other countries, polypharmacy in the elderly was found to be associated with female gender. Other known drivers of polypharmacy in the elderly include age, being separated/divorced/widowed, lack of education, higher body mass index, being bedridden and self-medication (Carmona-Torres et al., 2018). Factors associated with chronic polypharmacy involve similar characteristics, i.e., a more advanced age, female sex, living in an institution, chronic multimorbidity and multidose dispensing (Wastesson et al., 2019).

Among the factors contributing to polypharmacy, age is a particularly important one as polypharmacy prevalence rises dramatically with years of life. A clear example of such a correlation are the results of a nationwide Swedish study. Among those aged <60, 60–69, 70–79, 80–89 and over 90 years, polypharmacy was present in 8.5, 35.9, 54.8, 73.0 and 79.6%, respectively, with excessive polypharmacy peaking up to 36.4% in individuals aged 90 years and over (Zhang et al., 2020). Our observation of a mean age of 76 years among elderly patients on chronic polypharmacy in Poland corresponds well with this tendency.

A marked variation in geographical distribution of chronic polypharmacy among Polish elderly citizens was also an interesting finding of our analysis. No matter which administrative unit of the country is considered, Łódź appears to be the epicentre of chronic polypharmacy. There are other data proving that the health parameters in this region deviate negatively from national averages (World Health Organization, 2012). Perhaps the variation of polypharmacy prevalence in the elderly is not only a Polish specificity since similar findings were reported in Italy and the Netherlands (Franchi et al., 2013; Sinnige et al., 2016). Nevertheless, uneven distribution of chronic polypharmacy density across various regions should be taken into account when drawing up regional health plans.

Elderly patients on chronic polypharmacy characterised in our analysis were provided with care by multiple health professionals. This is certainly a reflection of their poor health and the need to obtain necessary health service. Nevertheless, these findings also indirectly point to a lack of coordinated care and imperfect communication between various prescribers and healthcare institutions, which leads to chronic polypharmacy in the elderly. So far, only several health conditions (e.g., myocardial infarction) have been covered by coordinated care in Poland, and when it comes to communication, the major innovative enabler ensuring it, i.e., the nationwide electronic health record, has not been fully introduced yet. Fortunately, both these solutions are included in short-term development plans of the National Health Fund and the Polish Ministry of Health. Therefore, it may be expected that this situation will change for better.

Not surprisingly, our cohort of the elderly people on chronic polypharmacy was found to be hospitalised twice more often than the general Polish population of citizens aged 65 years or over. Perhaps this correlation between polypharmacy and hospitalisations in elderly patients is not surprising as those with poorer health may not only need more drugs but also more frequent hospitalisations. However, it seems that hospitalisation is an independent risk factor for polypharmacy in general, and inappropriate polypharmacy in particular. In an Irish study conducted among older people admitted to hospital, the likelihood of potentially inappropriate prescriptions after admission was higher than prior to it (adjusted odds ratio 1.72), regardless of patients' characteristics (Pérez et al., 2018).

A specific local factor that may certainly have an impact on the rate of polypharmacy in Polish elderly people is the availability of basic drugs free of charge offered to this group of citizens. Starting from September 1, 2016, “*Program Leki 75+*” (“Drugs 75+ Program”) was initiated. It enabled those aged 75 years and above to obtain these drugs at no co-payment. The overall idea of the program was to ensure access to necessary medications to those at the highest risk of multimorbidity since, in general, the prescribed drugs are subject to co-payment by patients in Poland, which varies both across and within specific drug classes. This, however, might be an incentive to overprescribe both for prescribers and their patients rather than to look for other non-pharmacological options of addressing health problems. So far, this problem has not been extensively studied and dedicated research is required.

Finally, it needs to be emphasized that the analysed medicines that contribute to chronic polypharmacy are the drugs used typically for management of chronic conditions. Among the top five ones, four were indicated for lifelong treatment, i.e., 1. Metformin (mostly recommended for diabetes), 2. Atorvastatin (hyperlipidaemia, coronary artery disease), 4. Ramipril (hypertension, chronic heart failure and other cardiovascular conditions), and 5. Amlodipine (hypertension and other cardiovascular conditions). This list, undoubtedly grouping highly indicated drugs, differs considerably from the one including the most prevalent potentially inappropriate ATC codes identified in the Swiss elderly, of which the top five were Zolpidem, Estradiol, Acemetacin, Amiodarone and Trimipramine (Blozik et al., 2013). Thus, it might not be an easy task to discontinue such treatments in elderly patients in Poland without causing a serious disruption of their care.

Our findings also deserve practical solutions. A recent WHO report on polypharmacy urges countries to put polypharmacy high on their agendas in order to reduce its prevalence by implementing dedicated programs (World Health Organization, 2019). Quite often, however, it is still not the case in Europe. A search for polypharmacy management programs, undertaken within the framework of the SIMPATHY project, revealed such initiatives in five out of nine assessed countries only (McIntosh et al., 2018). Regrettably, no such official program was identified in Poland (Stewart et al., 2017), nor introduced to the date of this publication.

To be safe, effective and cost-effective, any program that attempts to deal with the complexities of prescribing medications in elderly people should be patient-centred, clinically robust, multidisciplinary and designed to fit into the healthcare system in which it is delivered (Stewart et al., 2017). Such a program may be based on one or multiple interventions. So far, various solutions have been proposed to reduce inappropriate prescribing and subsequent polypharmacy in older adults, ranging from comprehensive geriatric assessment, shared decision-making, medication reviews performed by either pharmacists or physicians, training of healthcare staff, use of various guidelines, checklists, up to different forms of computer and/or artificial intelligence-assisted clinical decision support systems (Lee et al., 2020).

Comprehensive approaches work well, e.g., dedicated palliative consultations resulted in a decrease in the number of drugs used in complex palliative patients in Poland (Grądalski, 2019). However, they also impose serious limitations related to their time-consuming nature, as well as a need for highly experienced staff. Unfortunately, with a very limited number of practicing geriatricians and absence of clinical pharmacists working in outpatient care, scaling up of such results has not been possible in Poland so far.

Considering the above, it seems to be much more realistic to implement simpler, explicit criteria-based interventions aimed at deprescribing, such as drug reviews using validated tools (e.g. STOPP/START or Beers criteria) (Kurczewska-Michalak et al., 2021). Such interventions may be further developed by dedicated applications or computer-assisted decision support systems, in order to promote their applicability. However, these approaches are still time-consuming, which makes their implementation a considerable challenge for busy clinicians who sometimes, unfortunately, do not pay much attention to the problem of polypharmacy.

In such circumstances, it may be expected that another intervention to be introduced soon will help prevention and management of polypharmacy in elderly patients in Poland. The Polish Act on the Profession of Pharmacists (Dziennik Ustaw, 2021) became effective in april 2021. Among innovations codified by this Act, there is a new service provided within pharmaceutical care which was not available in Poland before. Under the ministerial document specifying the scope of the service, it will cover identification, management and prevention of drug problems in general, and it will also include reviews of drugs which will most probably be reimbursed (Ministerstwo Zdrowia, 2021). Thus, access to professional drug reviews may be soon widely available in Poland, with obvious benefits for elderly people facing the risk of inappropriate polypharmacy.

This study has several limitations. One of them is that we were not able to correlate an individual exposure to polypharmacy with either the kind or number of conditions that an individual patient was diagnosed with. Similarly, we could not assess whether the identified cases met the criteria for either ‘appropriate’ or “inappropriate polypharmacy”. All that was not possible due to the fact that the nationwide electronic health record system has not yet been launched in Poland, which made comparisons between clinical and dispensation data very difficult and limited them to the reports on hospital stays or some outpatient services. Thus, we could only hypothesize that multimorbidity must have had an effect on polypharmacy prevalence in the studied population. There exists evidence proving that the greater the number of conditions an elderly patient is diagnosed with, the higher the probability of polypharmacy (Slabaugh et al., 2010).

Another limitation of our study is related to the scope of the analysed drugs which was narrowed down to reimbursed prescription medications only. In fact, polypharmacy is a problem that may be caused by various sorts of remedies, including non-reimbursed prescription drugs, as well as over-the-counter (OTC) drugs and dietary supplements which are often overused in Poland (Bochenek et al., 2016). Thus, our findings should be accepted as conservative estimates. A survey-based study assessing older (70+) primary care patients in Germany found that 26.7% of them were on prescribed polypharmacy, however, the percentage increased to 53.6% when OTC drugs were included as well (Junius-Walker Uet al, 2007). On the other hand, this is a natural disadvantage of analyses based on pharmacy claims data since they do not capture dispensation of OTC drugs or drug-similar products. However, the alternative approach, which is based on surveying and thus makes it possible to identify the use of non-prescription products, is subject to a large recall bias, and considerable underreporting.

Finally, our analysis covered only 1 year. In order to better understand the prevalence of polypharmacy in the elderly, and in particular, to observe its dynamics over time, the longitudinal analyses are warranted.

Nevertheless, this study has also a number of strengths. It was based on a nationwide database, with only a few drug groups of minor importance excluded from the analysis for practical reasons. Moreover, our analysis was based on dispensation, and not prescription data, which allowed us to avoid a considerable bias. Due to various reasons, a large number of prescriptions is never filled. We found this proportion to reach as many as 20.8% of all prescriptions issued in Poland (Kardas et al., 2020).

## Conclusion

This study was the first of this kind involving a nationwide assessment of chronic polypharmacy in Polish elderly people. We found that this problem affected one fifth of Polish older adults and it remains stable due to its direct relation to chronic conditions. We also observed a marked variation in geographical distribution of chronic polypharmacy in Polish elderly citizens, peaking in the Łódź Voivodeship. The medicines that contributed most to chronic polypharmacy in our analysis were the drugs used typically for management of chronic conditions. It is noteworthy, however, that our analysis did not cover OTC drugs which could impact polypharmacy even more. Considering the fact that the population of Poland is aging fast, and the number of elderly citizens is continuously increasing, one may expect a further rise in chronic polypharmacy among Polish elderly citizens in the upcoming decades. Therefore, our results confirm that this phenomenon is highly important for the national health policy and requires relevant interventions. The planned introduction of pharmaceutical care in Poland may be expected to provide a practical solution that will help to address this problem properly.

## Data Availability

The data analyzed in this study is subject to the following licenses/restrictions: The data that support the study findings are made available by the authors with the permission of NHF (data owner). Restrictions apply to the availability of the data which were used under the license for this study. Requests to access these datasets should be directed to przemyslaw.kardas@umed.lodz.pl.
